# Reconfiguring hot-hole flux via polarity modulation of p-GaN in plasmonic Schottky architectures

**DOI:** 10.1126/sciadv.adu0086

**Published:** 2025-03-07

**Authors:** Hyunhwa Lee, Yujin Park, Sanghee Nah, Mincheol Kang, Moonsang Lee, Jeong Young Park

**Affiliations:** ^1^ Department of Chemistry, Korea Advanced Institute of Science and Technology (KAIST), Yuseong-Gu, Daejeon 34141, Republic of Korea.; ^2^ McKetta Department of Chemical Engineering, University of Texas at Austin, Austin, TX 78712, USA.; ^3^ Seoul Center, Korea Basic Science Institute, Seoul 02841, Republic of Korea.; ^4^ Department of Materials Science and Engineering, Inha University, 100 Inha-ro, Michuhol-gu, Incheon 22212, Republic of Korea.; ^5^ Program in Semiconductor Convergence, Inha University, 100 Inha-ro, Michuhol-gu, Incheon 22212, Republic of Korea.

## Abstract

While energetic plasmonic hot carriers in nonthermal equilibrium states have pushed the limits of energy conversion efficiency in plasmon-driven photocatalysts and optoelectronics, the acceleration of plasmonic hot-hole flux remains a challenge. Here, we demonstrate an approach to control the generation and injection nature of plasmonic hot holes released from Au nanomesh/p-type GaN (p-GaN) Schottky architecture by modulating polarity of p-GaN. This polarity modulation enhances the flux of hot holes into the plasmonic platform, thereby accelerating Landau damping stemming from increased effective heat capacity of hot electrons in the metallic nanomaterial. We observed that this strategy drives the intensified hot-hole flux even in non–hot spot areas, hinting at the prospect of leveraging the complete potential of the plasmonic device beyond usual hot spots. The polarity modulation in plasmonic Schottky device gives rise to opportunities for manipulating the nature of plasmonic hot carriers for future energy conversion devices.

## Teaser

Polarity modulation in the Au/p-GaN Schottky structure boosts hot-hole flux by enhancing its generation and injection rates.

## INTRODUCTION

Plasmonic hot carriers generated via the nonradiative decay of localized surface plasmon resonance (LSPR) have great promise for photocatalytic and optoelectronic-based applications, such as photovoltaics, photodetectors, water-splitting, and artificial photosynthesis (*1*–*9*). Among them, hot holes have been deemed more suitable for efficient energy conversion compared to hot electrons, particularly in low photon energy regime (<1 eV), due to their asymmetrical energy distribution relative to the Fermi level despite the shorter mean-free path (MFP) and lifetime of hot holes (*10*–*12*). To fully capitalize on this potential, numerous inquiries have delved into extracting plasmonic hot holes over a tunable Schottky barrier at a metal-semiconductor (M–S) interface, aiming at efficient energy conversion applications (*13*–*16*). Obviously, for energy conversion with higher quantum efficiency, two essentials, the generation and injection of hot holes, are pivotal (*12*, *16*). However, to date, efforts to enhance hot-hole flux by tailoring their nature of generation and injection have primarily focused on optimizing the configurations or compositions of metallic nanomaterials, as well as their integration with semiconducting supports, such as GaN, NiO_x_, and Cu_2_O (*10*, *17*–*19*).

In plasmonic M–S Schottky architectures, achieving a high hot-hole density requires either increasing the extinction cross section in metallic nanostructures or optimizing the electronic band structure of the metal. Enhancing hot-hole injection, on the other hand, necessitates meticulous selection of metallic nanostructures or semiconductor materials to ensure proper Schottky barrier height, conservation of tangential-momentum across the interface, and a sufficiently long MFP of hot holes, thereby requiring careful selection of plasmonic materials and their pairing semiconductor supports. Myriads of experimental surveys have revealed that the hot-hole injection in a given plasmonic metal, when supported by different p-type semiconductors, is inevitably influenced by the unique electronic structures of these semiconductors, characterized by variations in refractive index, energy levels, and electron effective mass; this simultaneously alters the LSPR wavelength, Schottky barrier height, and moment mismatch. Consequently, these intertwined alterations concurrently affect the nature of hot-hole generation and injection, making it challenging to isolate and independently analyze their individual contributions. This complexity substantially complicates a clear understanding of the mechanisms underlying hot-hole flux enhancement. To elucidate the distinct contributions of hot-hole generation and injection, particularly their causal relationship to enhanced hot-hole flux in M–S Schottky architectures, it is crucial to precisely regulate the interwoven plasmonic environments. Therefore, an innovative model system is required to boost hot-hole flux while allowing for precise control over both hot-hole generation and injection, minimizing any unintended collective distortions in key plasmonic properties, such as LSPR wavelength, MFP of hot holes, Schottky barrier height, and near-field enhancement.

Here, we present a strategy to reconfigure plasmonic hot-hole flux emitted from Au nanomesh/p-type GaN (p-GaN) by manipulating the polarity environment of the semiconductor support. By maintaining a consistent compositional configuration in the plasmonic Schottky structure, our approach preserves key plasmonic properties, including LSPR wavelength, near-field enhancement, and Schottky barrier height, while specifically altering the hot-hole injection environment. We demonstrate that the plasmonic hot-hole flux is notably enhanced on Au nanomesh supported on polar c-plane p-GaN (c,p-GaN) substrate compared to nonpolar a-plane p-GaN (a,p-GaN) substrates, assisted by the presence of spontaneous polarization (P_SP_) aligning with the direction of hot-hole injection. Furthermore, we observe that this promoted hot-hole injection in the polar c,p-GaN architecture results in the reduced electron-phonon scattering times for hot electrons, as evidenced by ultrafast transient absorption analysis. Building on these findings of the enhanced hot-hole injection and quenched hot-electron dynamics in Au nanomesh/c,p-GaN, we propose that the facilitated hot-hole injection driven by P_SP_ contributes to accelerate LSPR dephasing, thereby effectively enhancing the hot-carrier generation rate, ultimately boosting hot-hole flux compared to the nonpolar a,p-GaN counterpart. Our analysis also unveiled that the polar p-GaN platform could inspire the highly amplified hot-hole injection in non–hot spot regions beyond hot spot areas. These findings highlight the potential for modulating the plasmonic hot-carrier behaviors and dynamics through prudent control of polarity of semiconductor support and offer an alternative approach to maximize plasmonic active areas in non–hot spot regions.

## RESULTS

### Characterization of photoelectrical properties of Au nanomesh/p-GaN Schottky structure

**Fig. 1.**
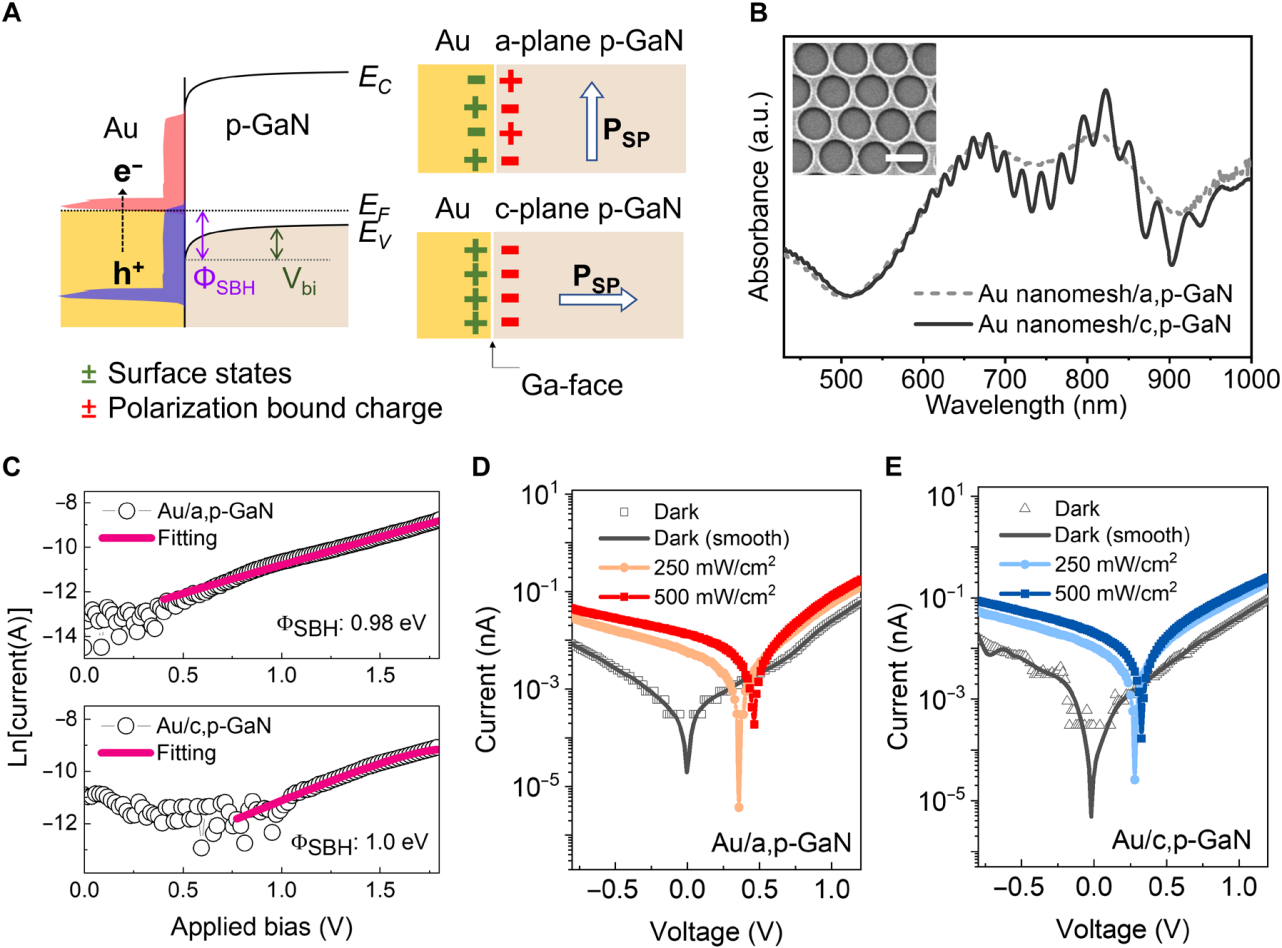
Plasmonic hot-hole flux on Au nanomesh/p-GaN Schottky junctions with different polarities of p-GaN. (**A**) Scheme of the energy band diagram of the Au/p-GaN Schottky structure with different polarities of p-GaN. An a,p-GaN indicates a nonpolar surface, while a c,p-GaN has arrays of dipole layers where negative charges preferentially accumulated, causing a strong *E* field at the interface. (**B**) Absorbance spectra of the Au nanomesh on both a,p-GaN and c,p-GaN. The inset shows a scanning electron microscopy image of the Au nanomesh. Scale bar, 500 nm. a.u., arbitrary unit. (**C**) *I-V* curves measured using pc-AFM system and the fit to the thermionic emission equation for Au nanomesh/a,p-GaN (top) and Au nanomesh/c,p-GaN (bottom). *I-V* curves measured on (**D**) Au nanomesh/a,p-GaN and (**E**) Au nanomesh/c,p-GaN under the excitation of 640 nm.

To investigate the influence of polarity modulation of the p-GaN substrate for plasmonic hot-hole flux, we constructed plasmonic architectures consisting of Au nanomesh/a,p-GaN and c,p-GaN, respectively. Figure 1A describes the energy band diagram of the Au nanomesh supported on p-GaN with different crystallographic planes. In this study, we chose p-GaN as an exemplary template for harvesting plasmonic hot holes through polarity modulation because GaN crystals exhibit P_SP_ depending on crystal growth orientation due to the lack of crystal inversion symmetry. Specifically, polar c plane (0001) shows a preferential accumulation of positive or negative charges and creates strong P_SP_, leading to large separation between electron and hole wave functions normal to *c* axis, while nonpolar a plane (11-20) has neutral charge distribution normal to out-of-plane (*20*). This unique inherent internal polarization in GaN offers the feasibility of tailoring internal electric field (*E* field) within the Schottky architecture, which gives rise to promote hot-hole injection. Therefore, polarity modulation of p-GaN enables clear insight into the nature of hot-hole injection efficiency by solely affecting hot-hole injection behavior while maintaining a consistent electronic structure (e.g., refractive index and Schottky barrier height) in the supporting semiconductor, thereby ensuring uniform LSPR wavelength and near-field intensities when paired with identical plasmonic nanostructures. In this context, we expect that the simultaneous termination of Ga and N species on the a plane indicates nonpolarity, while the nature of the Ga-faced c plane creates strong P_SP_ within the p-GaN substrate normal to *c* axis (*20*). This P_SP_ establishes an internal *E*-field at the interface between the Au nanomesh and c,p-GaN, aligned with the hot-hole flux direction. Consequently, plasmonic hot-hole transfer could be facilitated for Au nanomesh on c,p-GaN compared to that on a,p-GaN.

The a-plane and c-plane substrates were prepared by epitaxially growing Mg-doped p-GaN on different sapphire substrates with controlled orientations using metalorganic chemical vapor deposition (MOCVD). Their pure crystallinity and phase were verified through x-ray diffraction patterns, and both p-GaN substrates were confirmed to have a bandgap of approximately 3.4 eV, with negligible light absorption at visible spectrum (fig. S1). Subsequently, Au nanomesh was created on the p-GaN substrates as a plasmonic antenna using nanosphere lithography method, as depicted in fig. S2. The plasmonic Au nanomesh, with a thickness of 20 nm, was created by depositing Au films on top, filling the gaps of a plasma-etched polystyrene (PS) monolayer assembly. The absorption spectrum of Au nanomesh on both a-plane and c-plane substrates exhibited two distinct peaks centered at 660 and 810 nm (Fig. 1B), which were not observed on the bare p-GaN substrates. These peaks are considered plasmonic resonance absorption peaks within Au nanomesh, stemming from the geometric shape of nano-hole arrays (*21*, *22*). The perceived interference fringes of Au nanomesh/c,p-GaN arise from the superposition of multiple reflections within the c,p-GaN substrate (fig. S1B). Given the large bandgap of 3.4 eV for both a,p-GaN and c,p-GaN and their low visible light absorption intensities, light absorption in the visible to near-infrared region is governed by light absorption within the plasmonic Au nanomesh. The plasmon-driven hot-hole flux was detected as a steady-state current by depositing a Ni/Au alloy thin film on the p-GaN substrate as an ohmic electrode (*23*), thereby constructing a global circuit for the Au nanomesh/p-GaN Schottky structures. The resistance of the Ni/Au alloy ohmic electrode on both a,p-GaN and c,p-GaN was assessed to be 100 ± 0.5 kilohms, indicating negligible differences in ohmic loss (fig. S3). Since the hot-hole flux is proportional to the number of hot holes surpassing the Schottky barrier, it is crucial to determine the barrier heights of Au nanomesh on a,p-GaN and c,p-GaN substrates. To this end, photoconductive atomic force microscopy (pc-AFM) measurements were used to analyze nanoscopic current-voltage (*I*-*V*) curves on Au nanomesh/p-GaN structures (Fig. 1C). The pc-AFM uses a cantilever connected to the ohmic pad of p-GaN, scanning along the surface of Au nanomesh, thus capable of detecting hot-hole flux as currents with high spatial resolution (*24*, *25*). The measured *I*-*V* curves were fitted to the thermionic emission equation (*26*, *27*) to evaluate the Schottky barrier heights (Φ_SBH_), as shown in table S1. Accordingly, the Schottky barrier heights were determined to be 0.98 and 1.0 eV for Au nanomesh on nonpolar a,p-GaN and polar c,p-GaN, respectively. These comparable barrier heights observed on Au nanomesh on both a,p-GaN and c,p-GaN substrates suggest that the distinct internal dipole arrangements of p-GaN induced by different growth orientations have minimal impact on the threshold energy required to initiate hot-hole transfer. Subsequently, the steady-state plasmonic hot-hole flux was characterized by measuring the *I*-*V* curves on Au nanomesh/p-GaN substrates under 640-nm excitation (Fig. 1, D and E). In the control experiment conducted on bare p-GaN substrates, we confirmed that *I*-*V* curves measured on both bare a,p-GaN and c,p-GaN substrates exhibit currents that are four orders of magnitude lower than those measured on the Au nanomesh surface, without any photoresponse (fig. S4). Both Au nanomesh/a,p-GaN and Au nanomesh/c,p-GaN structures demonstrate progressively increased short-circuit current (*J*_sc_) and open-circuit voltage (*V*_OC_) with increased light intensity (fig. S5). Notably, we confirmed twofold increased hot-hole–driven photocurrent on Au nanomesh/c,p-GaN compared to Au nanomesh/a,p-GaN under the given light intensity. Incidentally, the *V*_OC_ was observed to be lower on Au nanomesh/c,p-GaN structure, indicative of the reduced built-in potential (*V*_bi_). Since the *V*_bi_ is defined as work function difference between Au nanomesh and p-GaN substrate, the preferential negative charge accumulated on the c,p-GaN substrate results in an elevated Fermi level of p-GaN, thereby lowering *V*_bi_ compared to Au nanomesh/a,p-GaN. Considering that Au nanomesh on both a,p-GaN and c,p-GaN have comparable light absorbance intensity and the Schottky barrier heights, the enhanced hot-hole–driven photocurrent on Au nanomesh/c,p-GaN can be attributed to the strong P_SP_ created within the p-GaN substrate. In particular, the negative charge distribution on the surface of c,p-GaN establishes a positively charged image force within the Au nanomesh and generates a Coulombic attraction that facilitates more efficient hot-hole injection toward c,p-GaN substrates, compared to the nonpolar a,p-GaN.

### Modulation of ultrafast hot-carrier dynamics driven by polarity of p-GaN

**Fig. 2.**
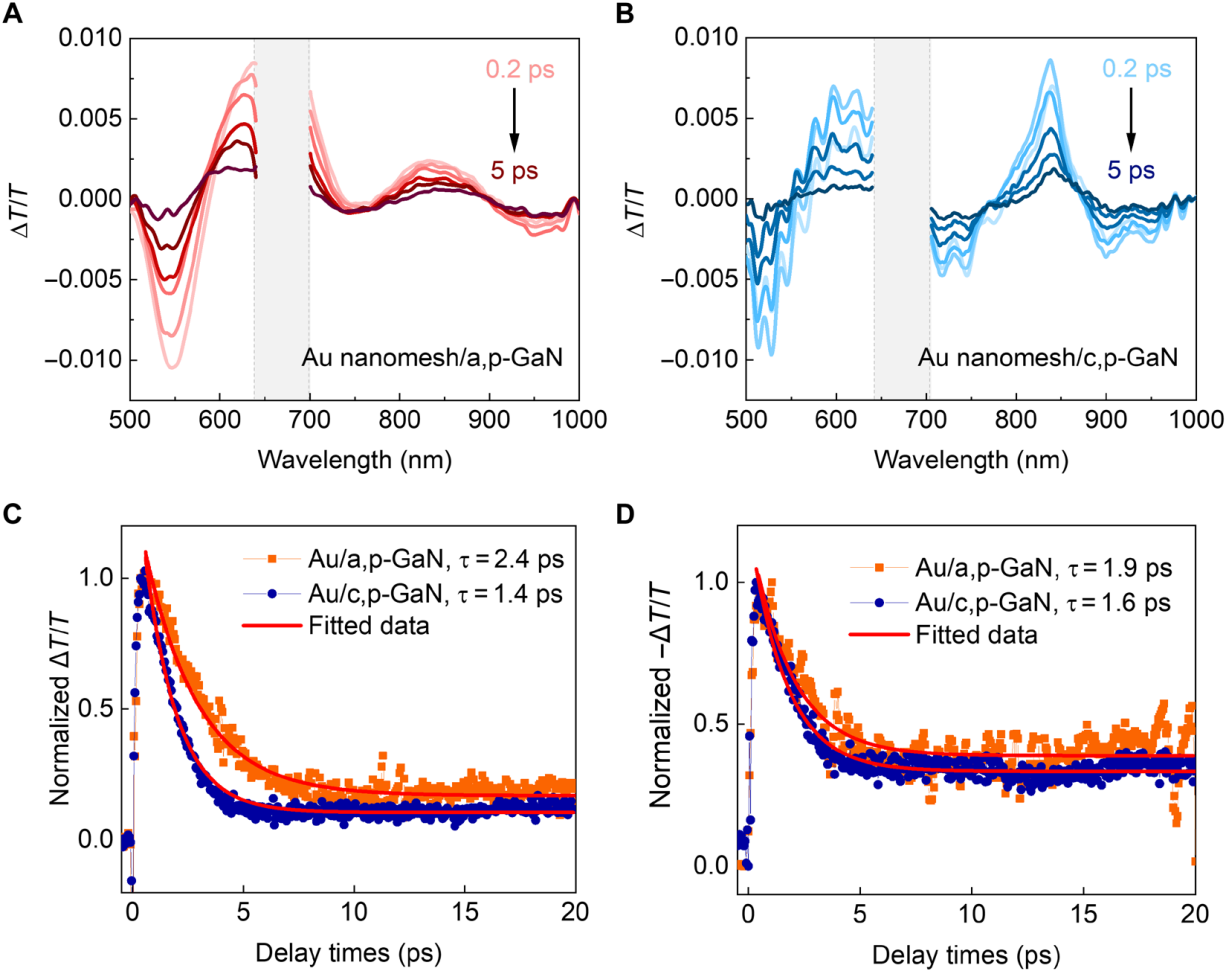
Modified hot-electron dynamics on Au nanomesh/p-GaN with different internal polarizations of the p-GaN. Transient absorption spectra of (**A**) Au nanomesh/a,p-GaN and (**B**) Au nanomesh/c,p-GaN excited at 610 nm with a power of 50 μWcm^−2^. Decay profiles of Au nanomesh on a,p-GaN and c,p-GaN substrates probed at (**C**) 610 nm and (**D**) 968 nm.

Subsequently, we conducted femtosecond transient absorption analysis on plasmonic Au nanomesh/p-GaN with different orientations to investigate the effects of enhanced hot-hole injection on hot-carrier dynamics. The transient absorption spectra of Au nanomesh/a,p-GaN and Au nanomesh/c,p-GaN excited at 610 nm are shown in Fig. 2 (A and B, respectively). The fringes observed in the Au nanomesh/c,p-GaN structure were originated from Fabry-Pérot interference, causing multiple reflections within the substrate (*15*). Regardless of the interference fringes, both structures exhibit strong bleaching peaks aligning with the LSPR absorption peaks along with three winglets around the bleaching peaks. Control experiments conducted on bare p-GaN substrates displayed neglectable spectral features across the visible spectrum, confirming that the bleaching peaks and winglets are not related to any excitations within p-GaN substrates (fig. S6). The observed bleaching peaks arise from the depletion of ground state carriers, while the winglets come from the absorption of hot carriers within the plasmon band (*28*, *29*). We observed a higher optical density for the Au/c,p-GaN at the bleaching peak located at 810 nm, which is associated with higher steady-state absorption. Therefore, we selected the bleaching peak centered at 610 nm to probing hot-electron dynamics with comparable hot-electron densities. The lifetime of plasmonic hot electrons was examined by tracing the recovery of the bleaching peak centered at 610 nm and fitting it to single-exponential decay (Fig. 2C). The hot-electron lifetimes were observed to be 2.4 ps for Au nanomesh/a,p-GaN and 1.4 ps for Au nanomesh/c,p-GaN, which are governed by the electron-phonon scattering. Notably, hot electrons decay faster for Au nanomesh on polar c,p-GaN substrate, where hot-hole injection is effectively promoted, compared to that on nonpolar a,p-GaN template. The faster hot-electron relaxation was consistently observed at the decay profile probed at the lowest-energy winglet located at 968 nm, showing 1.9 and 1.6 ps for Au/a,p-GaN and Au/c,p-GaN, respectively (Fig. 2D). These results align with expectations for the M–S Schottky structure, where hot-hole injection leads to a reduced electron-phonon coupling time due to the limited electronic temperature resulting from the increased electronic heat capacity (*30*). Moreover, the increased electronic heat capacity resulting from promoted hot-hole injection offers an opportunity to accelerate Landau damping, rendering faster hot-carrier generation rate and, consequently, an increased hot-hole flux, as discussed in detail further down. Therefore, these findings, which show the rapid decay of hot electrons within plasmonic Au nanomesh induced by promoted hot-hole injection on the polar p-GaN substrate, highlight that both hot-carrier transfer and generation rates can be modulated by the polarity of the p-GaN substrate.

### Direct visualization of modified hot-hole flux driven by p-GaN polarity

**Fig. 3.**
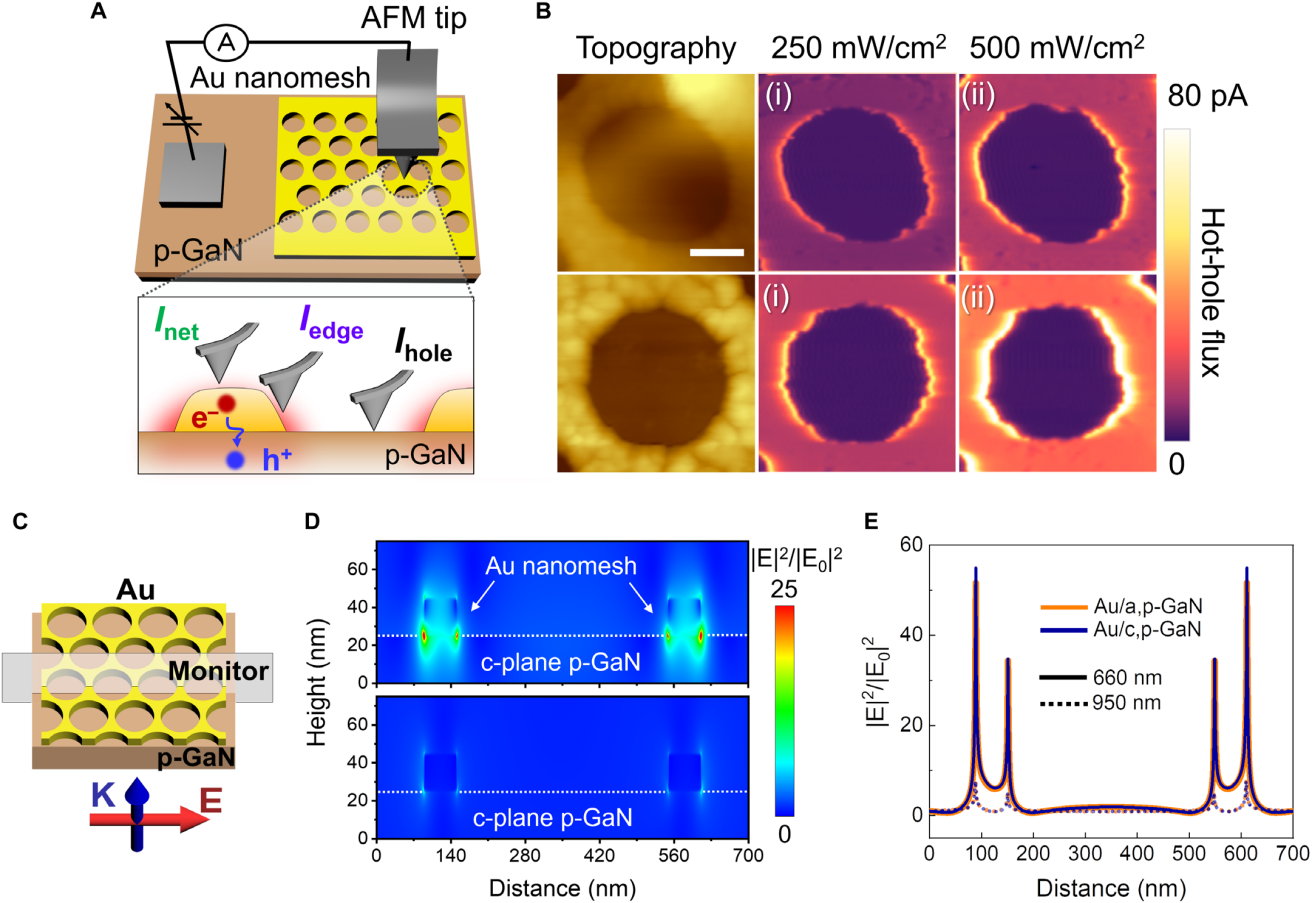
Direct visualization of modified hot-hole flux on plasmonic Au nanomesh/p-GaN with different internal polarizations of the p-GaN. (**A**) Schematic illustration showing the probing of hot-hole flux on the Au nanomesh/p-GaN using pc-AFM system. Upon illumination, plasmonic hot carriers are generated, subsequently injecting hot holes into the p-GaN substrate. (**B**) Topography and photocurrent maps of Au nanomesh on a,p-GaN (top images) and on c,p-GaN (bottom images). Photocurrent maps were measured under 640 nm with the intensities of [(B), i] 250 mW/cm^2^ and [(B), ii] 500 mW/cm^2^. Scale bar, 150 nm. (**C**) Scheme depicting the FDTD simulation conditions under light incidence of linearly polarized light in the in-plane direction. (**D**) Cross-sectional *E*-field distribution on Au nanomesh/c,p-GaN under 660 nm (top image) and 950 nm (bottom image). (**E**) *E*-field profile of Au/a,p-GaN and Au/c,p-GaN obtained along the white dashed line in Fig. 3D.

To further elucidate the local evolution of hot-hole flux within the Au nanomesh/p-GaN, we measured the spatial distribution of photocurrents with nanometer-scale resolution using pc-AFM (Fig. 3A). During the acquisition of photocurrent maps on the Au nanomesh/p-GaN structures, the plasmonic platforms were excited by the linearly polarized light in the in-plane direction at a wavelength of 640 nm. Figure 3B displays photocurrent maps scanned on a single hole within Au nanomesh deposited on a,p-GaN and c,p-GaN, at various light intensities. Upon scanning the surface of the Au nanomesh, we observed clear photocurrent contrast across various local positions on the Au nanomesh. The highest photocurrent was detected at the edge (*I*_edge_), followed by a moderate photocurrent recorded at the net (*I*_net_), but the hole area (*I*_hole_) exhibited an insignificant photocurrent. In addition, both *I*_edge_ and *I*_net_ increased their photocurrent in proportion to the incident light intensity, whereas *I*_hole_ displayed negligible photoresponse. Overall photocurrent in c-plane template exhibited superior values over the entire laser intensities and scanning regions, compared to those of a-plane architecture. Compared to the Au nanomesh/a,p-GaN structure, the Au nanomesh/c,p-GaN exhibits an increase in photocurrent of 1.7 (1.68) times at *I*_edge_, 2.21 (2.09) times at *I*_net_, and an average increase of 2.38 (2.14) times across the entire illuminated area under intensities of 250 mW/cm^2^ (500 mW/cm^2^).

To shed light on how the local contrast of photocurrent is affected by the *E*-field distribution in the LSPR environment, we implemented finite-difference time-domain (FDTD) computations. In the simulation, linearly polarized light was directed through the sequence of p-GaN and Au nanomesh using the same setup as in pc-AFM (Fig. 3C). We confirmed the strong *E*-field confinement created at the edges of the Au nanomesh under resonance wavelength (660 nm), which was not shown under nonresonance wavelength (950 nm), in both Au nanomesh/a,p-GaN and Au nanomesh/c,p-GaN structures (Fig. 3D and fig. S7). Figure 3E shows the profile of *E*-field distribution along the white dashed line in Fig. 3D. The hot spots are clearly visible at the edges of the Au nanomesh, indicating an intensified *E* field with a broader distance under 660 nm compared to 950 nm. Therefore, the substantially intensified plasmonic hot-hole flux at the edges of the Au nanomesh on p-GaN substrates manifests the activation of plasmonic hot spots.

**Fig. 4.**
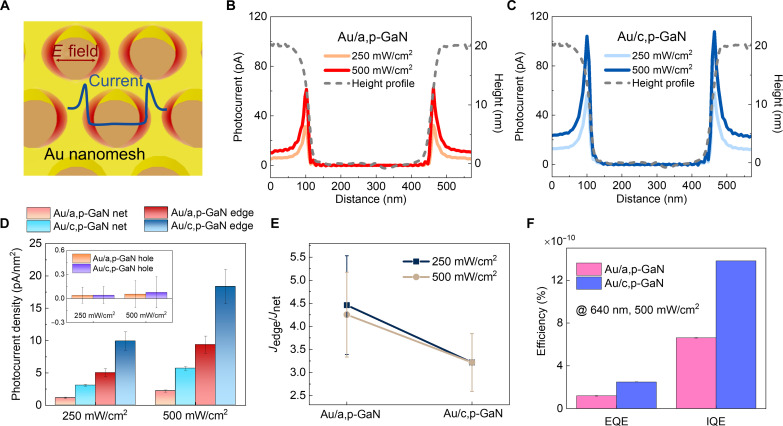
Quantitative analysis of nanoscopic hot-hole flux depending on the interfacial polarity of Au/p-GaN Schottky structures. (**A**) Schematic illustrating the distribution of current across the surface of Au nanomesh/p-GaN, correlated with the distribution of the *E* field at the hot spots. Current profile measured across the surface of Au nanomesh deposited on (**B**) a,p-GaN and that on (**C**) c,p-GaN substrate. The photocurrents appear to be at maximum at the regions aligned with the edges of the Au nanomesh. (**D**) Comparison of local photocurrent densities observed at the net, edge, and hole regions of Au nanomesh/p-GaN structures. (**E**) Current density ratio between edge (*J*_edge_) and net (*J*_net_) regions on the Au/a,p-GaN and Au/c,p-GaN structures. (**F**) External quantum efficiency (EQE) and internal quantum efficiency (IQE) of Au/p-GaN structures at the wavelength of 640 nm with an intensity of 500 mW/cm^2^.

As the hot spots contribute to amplifying plasmonic hot-hole flux, we anticipate that the peak value of photocurrent will be positioned at the edges of the Au nanomesh, with mild photocurrent observed at the net, as depicted in Fig. 4A. To further interrogate the influence of P_SP_ of p-GaN substrates on hot-hole flux, we extracted the photocurrent profiles from the photocurrent maps measured on Au nanomesh/a,p-GaN and Au nanomesh/c,p-GaN in Fig. 4 (B and C, respectively). The height profile of the Au nanomesh is overlaid with the photocurrent profiles to provide a clear understanding of how the photocurrent evolves with respect to the distinct positions of the Au nanomesh. We confirmed that the photocurrent profiles of both Au nanomesh/a,p-GaN and Au nanomesh/c,p-GaN structures are well aligned with their *E*-field distribution, showing intensified photocurrents at the edges. Notably, the photocurrent of the Au nanomesh on c,p-GaN was observed to be higher than that on a,p-GaN for a given light intensity. Considering the potential variability in contact area between the AFM tip and the Au nanomesh depending on the scanning spot, for example, an increase in contact area at the edge site and a decrease at the net site, we meticulously assessed the contact area at various scanning positions (table S2) (*31*). As anticipated, the contact area at the edge site was approximately 1.2 times larger than that at the net site on both Au nanomsh/a,p-GaN and Au nanomesh/c,p-GaN structures. While the contact area at the edge increased by 1.2 times compared to the net, the photocurrent increased by approximately a factor of 4. Therefore, we believe that, in addition to the increased contact area, confined-surface plasmons encircling the holes in the Au nanomesh considerably contribute to the reinforcement of photocurrent at the edge. In other words, the LSPR-driven confined interaction between the incident light and the Au nanostructure enhances light absorption at the edges, marking a substantial improvement over the nonresonant region. Consequently, the LSPR-driven hot holes can be efficiently funneled over the Schottky barrier, guided by the precise geometric control of the plasmonic structure.

### Quantification of modified hot-hole flux induced by polarity modulation of p-GaN

To quantitatively investigate the modulation of hot-hole flux on Au nanomesh/p-GaN with different polarities of p-GaN, we compare the local photocurrent density at various positions, including the edge (*J*_edge_), the net (*J*_net_), and the hole (*J*_hole_,) regions of the Au nanomesh supported on a,p-GaN and c,p-GaN substrates by normalizing photocurrent to the contact area (Fig. 4D). Compared to the Au nanomesh/a,p-GaN structure, the Au nanomesh/c,p-GaN demonstrates an increase in current density by 1.97 (1.96) times at the edge (*J*_edge_), 2.73 (2.59) times at the net (*J*_net_), and 2.37 (2.13) times on average across the entire area under illumination intensities of 250 mW/cm^2^ (500 mW/cm^2^). Given the comparable intensity of *E*-field confinement at hot spots in both structures (Fig. 3E), the increased hot-hole flux in Au/c,p-GaN is attributed to facilitated hot-hole injection driven by the P_SP_ of the polar c,p-GaN, whose dipole direction aligns favorable with the hot-hole transfer. Furthermore, we analyzed the ratio of *J*_edge_ and *J*_net_ for both Au nanomesh/a,p-GaN and Au nanomesh/c,p-GaN to discern the regions most affected by the P_SP_ of the p-GaN substrate (Fig. 4E). Notably, the Au nanomesh/c,p-GaN exhibits a lower ratio of 3.2 across all light intensities compared to that of Au/a,p-GaN, which is 4.5 and 4.3 for 250 and 500 mW/cm^2^, respectively. These findings indicate that the increase in *J*_net_ is more prominent compared to that of *J*_edge_ when transitioning from nonpolar a,p-GaN to polar c,p-GaN substrates, highlighting that the inherent P_SP_ in c,p-GaN has a substantial impact on hot-hole flux (*J*_net_) in non–hot spot regions, compared to the *J*_edge_ in the hot spot area. In addition, the slope of *J*_edge_/*J*_net_ was mitigated with increased light intensity, suggesting that the P_SP_-induced J_net_ enhancement becomes even more pronounced with higher light intensity. In other words, the strong P_SP_ of c,p-GaN is crucial, as it can substantially enhance plasmon-driven photocurrent even in non–hot spot areas. The external quantum efficiency and internal quantum efficiency were further examined (see note S1) and found to be 2.03 times higher on Au nanomesh/c,p-GaN compared to Au nanomesh/ a,p-GaN (Fig. 4F), supporting the promoted hot-hole transfer on c,p-GaN resulting in enhanced solar conversion efficiency.

### Mechanism of plasmonic hot-carrier flux reconfiguration via polarity modulation of p-GaN

**Fig. 5.**
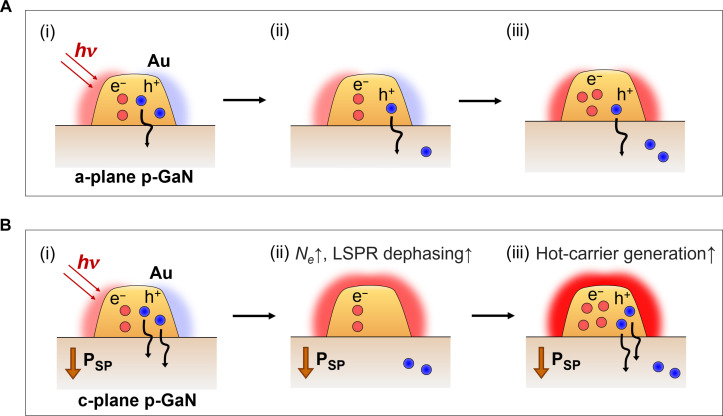
Comparative dynamics of hot-carrier generation and injection in Au nanomesh depending on p-GaN polarity. Schematic comparison of hot-hole dynamics in (**A**) Au nanomesh/a,p-GaN and (**B**) Au nanomesh/c,p-GaN structures. (i) LSPR excitation in the Au nanomesh generates hot carriers through Landau damping, followed by the injection of hot holes into GaN substrate. (ii) In polar c,p-GaN, the P_SP_ field enhances hot-hole injection, resulting in a higher effective hot-electron density (*N_e_*) in the Au nanomesh and accelerated LSPR dephasing compared to nonpolar a,p-GaN. (iii) Consequently, this increased LSPR dephasing rate boosts hot-carrier generation rate, leading to a higher hot-hole flux over time in c,p-GaN compared to a,p-GaN.

To give a clear picture of the influence of internal polarization of p-GaN on the hot-hole flux, Fig. 5 illustrates the mechanism of hot-carrier generation and injection within an Au nanomesh/p-GaN Schottky structure with different p-GaN polarities. When LSPR occurs on the Au nanomesh, free electrons vibrate coherently with the incident light. These oscillating electrons then transfer energy to produce hot carriers through momentum matching from the localized *E* field, a process known as Landau damping. This is enabled by the large wave vector (Δ*k*) presenting in the localized *E* field, resulting from the high Fermi velocity (υ*_F_*) compared to free space (*32*). Subsequently, hot holes with sufficient energy to surpass the Schottky barrier height are injected into the p-GaN. More efficient hot-hole injection is achieved in the polar c,p-GaN substrate, compared to the nonpolar a,p-GaN, due to the established P_SP_. This enhanced hot-hole injection in c,p-GaN leads to higher effective hot-electron density (*Ne*) than in a,p-GaN. Considering the rate of LSPR, decay through Landau damping (γ*_LD_*) is defined as γ*_LD_* = (3/8)×(υ*_F_*/*d_eff_*), where *d_eff_* is the spatial extent of the field (*33*, *34*), the increase in *Ne* contributes to an effectively increased υ_*F*_ and, thus, promotes LSPR dephasing. Therefore, the accelerated LSPR dephasing on Au nanomesh/c,p-GaN increases the hot-carrier generation rate, resulting in a higher hot-hole flux over time, compared to that of Au nanomesh/a,p-GaN. Incidentally, the increased *Ne* leads to an elevated electronic heat capacity in the Au nanomesh, thereby mitigating the hot electron temperature established by electron-electron scattering after Landau damping. This reduced hot electron temperature is supported by the observed decrease in transient absorption intensity at the plasmonic bleaching peak (610 nm) for Au/c,p-GaN compared to Au/a,c-GaN (Fig. 2, A and B). The promoted LSPR dephasing on the Au/c,p-GaN is not confined to the hot spot but occurs at non–hot spot regions as well, which is evidenced by overall elevated photocurrent across the net regions, in contrast to the minimal increment in *I*_net_ on the Au/a,p-GaN (Fig. 3B). We attribute the enhanced hot-hole flux in the c-plane platform to the accelerated hot-hole injection induced by P_SP_ across the entire plasmonic metal structure, leading to increased surface collisions and LSPR dephasing throughout the plasmonic architecture. This acceleration promotes the hot-hole generation, allowing for incremental photocurrent beyond the hot spot regions in the polar template.

## DISCUSSION

In conclusion, we have demonstrated that modulating the polarity of p-GaN enhances plasmonic hot-hole flux in Au nanomesh/p-GaN Schottky structures. Specifically, the establishment of P_SP_ within polar c,p-GaN promotes hot-hole injection, leading to an approximately two-fold increase in hot-hole flux, compared to nonpolar a,p-GaN counterpart. The impact of P_SP_ within p-GaN templates was further investigated by acquiring a landscape of hot-hole flux distribution on Au nanomesh/p-GaN structures with high spatial resolution using pc-AFM. We observed pronounced hot-hole flux at the hot spots situated in the edge sites, while the P_SP_-driven hot-hole injection was more facilitated across the net region of non–hot spot areas. This pronounced enhancement of hot-hole flux in non–hot spot areas allows the full potential to maximize the hot-hole flux beyond usual hot spots. In addition, we demonstrated that this facilitated hot-hole injection reduces hot-electron lifetime by increasing effective hot-electron density and electronic heat capacity, thereby accelerating electron-phonon scattering within the Au nanomesh. Building on these comprehensive examinations using pc-AFM and ultrafast spectroscopy, we propose that enhancing hot-hole injection by controlling the internal polarization of p-GaN facilitates LSPR dephasing and improves the hot-carrier generation rate. These insights into hot-carrier transport and dynamics at extreme spatial and temporal limits underscore the substantial influence of polarity modulation in semiconductor supports on hot-hole generation and injection processes, as well as their causal relationship in enhancing hot-hole flux. Moreover, the polarity of p-GaN offers a promising strategy to tailor hot-carrier injection and generation efficiency, which is essential for rational design of plasmonic devices with high quantum efficiency.

## MATERIALS AND METHODS

### Preparation of GaN templates

p-GaN layers with a c- and a-plane orientation were grown on 2-inch (5.08 cm) c-plane (0002) and r-plane (1–102) sapphire substrates using Aixtron G3 2600 MOCVD. Trimethylgallium and ammonia (NH_3_) gases acted as precursor materials for Ga and N elements, respectively. Bicyclopentadienyl-magnesium (Cp_2_Mg) was introduced as a source of p-type doping. Before the growth of the p-doped GaN films, the contaminants on the substrate underwent thermal cleaning at 1150°C for 5 min under H_2_ ambient in the MOCVD reactor. After that, 4-μm-thick undoped GaN buffer layers were deposited at 1050°C under N_2_ ambient, succeeded by the growth of 380-nm-thick Mg-doped p-GaN films with a doping concentration of 5 × 10^18^ cm^−3^. The electrical activation of the templates was conducted at 750°C under N_2_ ambient for 1 min using rapid thermal annealing. For ohmic contacts, Ni (10 nm) and Au (10 nm) films were sequentially deposited using e-beam evaporator, followed by annealed at 480°C for 70 min before fabricating Au nanomesh structure.

#### Fabrication of Au nanomesh on p-GaN by nanosphere lithography technique

Au nanomesh on p-GaN was created using nanosphere lithography method, as described elsewhere. A PS latex solution dispersed 10 wt % in water (Sigma-Aldrich Inc.) was diluted with ethanol with 1:1 v/v %. Two hundred microliters of 2% SDS aqueous solution was drop-casted on the surface of a water vessel, followed by oblique immersion of a slide glass into the water. Twenty microliters of the PS solution was dispensed along the surface of the slide glass. Prepared p-GaN substrate was gently immersed into the water vessel. Once the dispensed PS assembled into a monolayer, the immersed p-GaN substrate was lifted to scoop up the floating PS monolayer on the water surface. After completely drying the p-GaN substrate with air flow, reactive ion etching process was conducted under O_2_ atmosphere with 80 W for 1 min to reduce the size of PS beads. A gold film with 20-nm thickness was deposited on the substrate at the rate of 1 Å/s. By removing the PS monolayer with ultrasonication, the patterned Au nanomesh was created on the p-GaN (see fig. S2 in the Supplementary Materials). The patterned Au nanomesh was confirmed to be uniformly smooth on both a,p-GaN and c,p-GaN substrates, exhibiting comparable roughness values of 1.35 ± 0.21 nm for a,p-GaN and 1.43 ± 0.21 nm for c,p-GaN, respectively.

#### pc-AFM measurement

pc-AFM was constructed by modifying the commercial AFM system (Agilent 5500), where the sample stage was modified with a waveguide, consisting of right-angled fused silica prism and a lens, for a laser source (OBIS series, Coherent). The laser was directed perpendicular to the backside of the sample. An external current preamplifier (model SR560, SRS) was used to form an electric circuit to collect the current, which was connected between PtIr-coated AFM probe (PPP-CONTPt, Nanosensors) and the ohmic pads on the sample. During scanning, the hot-hole flux was collected as a photocurrent signal through the external circuit between the probe and ohmic pad. To ensure stable and repeatable photocurrent map acquisition without distortion or structural damage, the tip load was set to 0.02 nN.

#### FDTD simulations

An FDTD method (FDTD Solutions, Lumerical) was used to calculate *E*-field distributions of the Au nanomesh/p-GaN. A linearly polarized Gaussian field was used as the incident light, with a wavelength range from 400 to 1000 nm. The dielectric function of gold was adopted from the Johnson and Christy’s parameters (*35*), while the refractive indices of GaN substrate were taken from a previous reference (*36*). A mesh with 5-nm spacing covered the Au nanomesh on p-GaN.

#### Ultrafast transient absorption spectroscopy

The hot electron dynamics in the Au nanomesh across the visible spectrum were investigated using femtosecond time-resolved transient absorption (TA) microscope (ST015) at Korea Basic Science Institute. A Yb:KGW regenerative amplifier (Light Conversion, PHAROS) generating pulses at 100 kHz was used to generate the pump (650 nm) and probe (500 to 1000 nm) beams. These beams were combined via a dichroic beam splitter, and the collinearly propagating beams were focused onto the Au nanomesh using a reflective objective [numerical aperture (NA) 0.65]. The transmitted probe beams were collected with an objective lens (NA 0.4) and directed toward a silicon-based array detector. Modulated probe transmission spectra, with and without the pump beam at 40 Hz, were recorded using the Harpia-TA system (Light Conversion).

## Supplementary Materials

### This PDF file includes:

Figs. S1 to S7 Supplementary Note 1 Tables S1 and S2
