# Supplementary material for: Reconfiguring hot-hole flux via polarity modulation of p-GaN in plasmonic Schottky architectures
Source: Sci Adv. 2025 Mar 7;11(10):eadu0086. doi: 10.1126/sciadv.adu0086 (PMC11887797; doi:10.1126/sciadv.adu0086)
Supplement: Supplementary file 1 — Figs. S1 to S7 Supplementary Note 1 Tables S1 and S2 [file sciadv.adu0086_sm.pdf]

Science Advances
AAAS

Supplementary Materials for

Reconfiguring hot-hole flux via polarity modulation of p-GaN in plasmonic Schottky architectures

Hyunhwa Lee *et al.*

Corresponding author: Moonsang Lee, mslee@inha.ac.kr; Jeong Young Park, jeongypark@kaist.ac.kr

Sci. Adv. **11**, eadu0086 (2025)
DOI: 10.1126/sciadv.adu0086

This PDF file includes:

Figs. S1 to S7
Supplementary Note 1
Tables S1 and S2

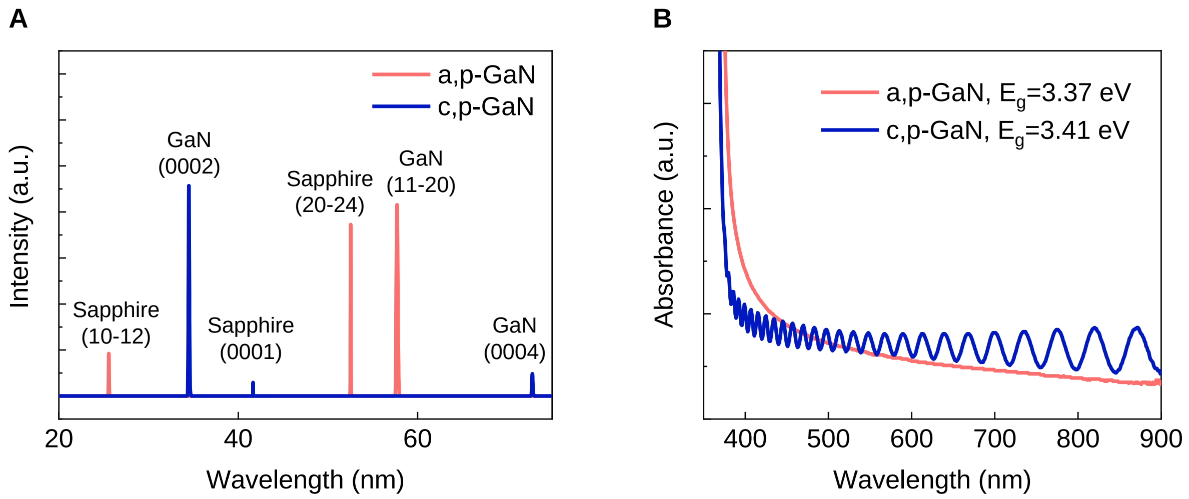

Fig. S1. Structural and optical properties of GaN substrates. (A) X-ray diffraction (XRD) patterns and (B) absorbance spectrum of a,p-GaN and c,p-GaN substrates.

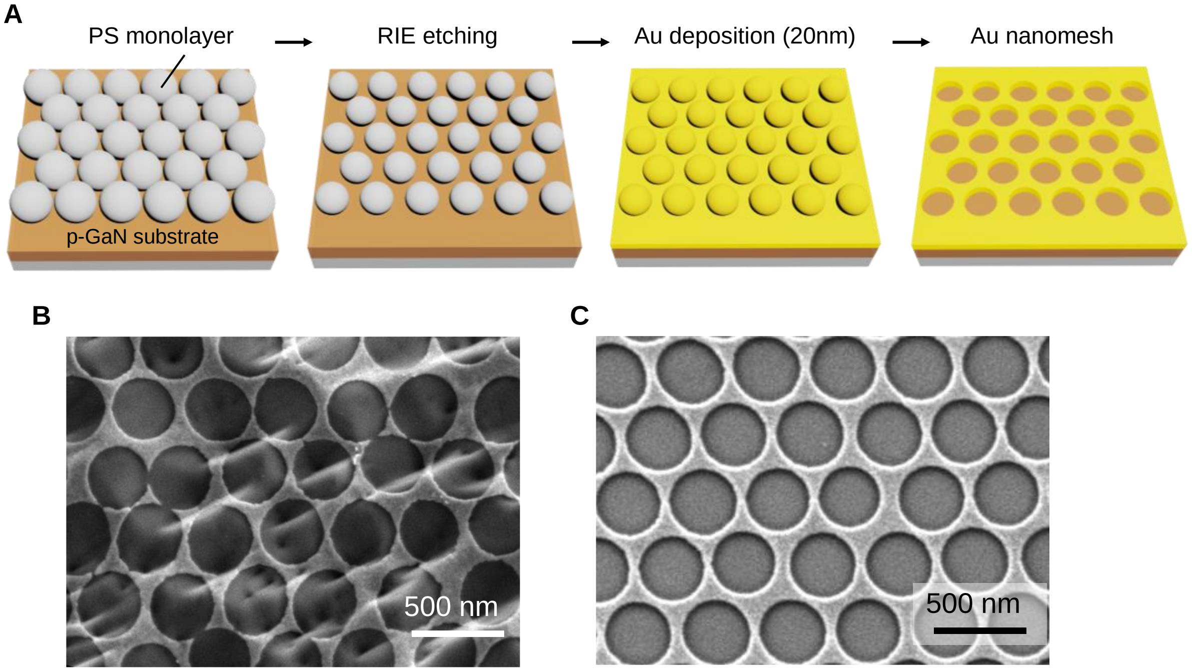

Fig. S2. Fabrication process of Au nanomesh. (A) Schematic of the process for patterning Au nanomesh on a p-GaN substrate using nanosphere lithography technique. Polystyrene (PS) nanobeads with a diameter of 460 nm were assembled into a monolayer on p-GaN substrate. The size of the nanobeads were then reduced through a reactive ion etching (RIE) process with oxygen gas. Subsequently, a 20 nm of Au thin film was deposited on top of the etched PS monolayer. The PS nanobeads were then removed by ultrasonication. SEM images showing the Au nanomesh on (B) nonpolar a,p-GaN and (C) that on polar c,p-GaN substrate.

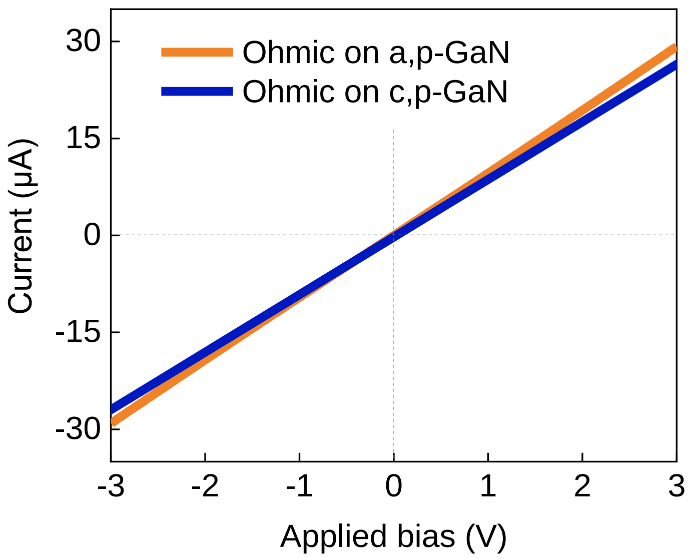

Fig. S3. *I–V* curves measured between Ni/Au alloy ohmic contact and p-GaN substrates.

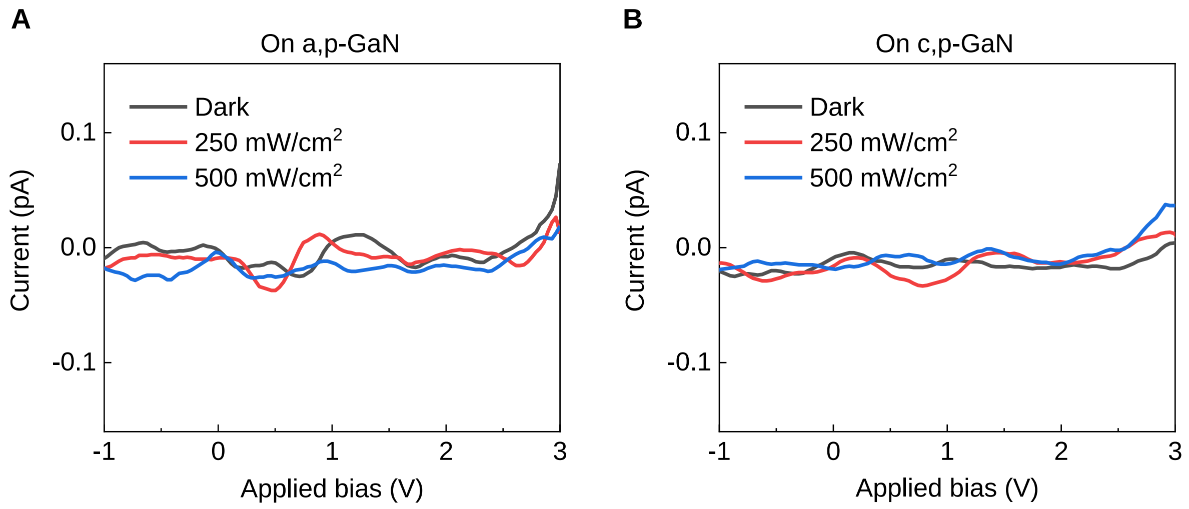

Fig. S4. Photoelectrical properties of bare p-GaN substrates. *I*–*V* curves measured (A) a bare a,p-GaN and (B) a bare c,p-GaN substrate using pc–AFM system.

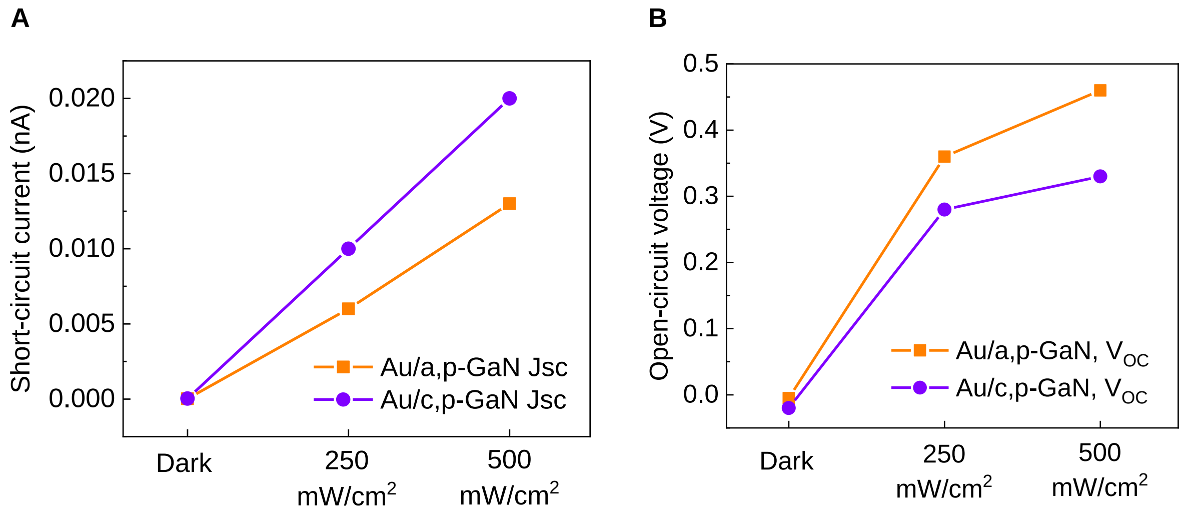

Fig. S5. Photoelectrical properties of Au nanomesh/p-GaN structures. (A) Short-circuit current (J_{SC}) and (B) open-circuit voltage (V_{OC}) measured on the Au nanomesh/a,p-GaN and Au nanomesh/c,p-GaN.

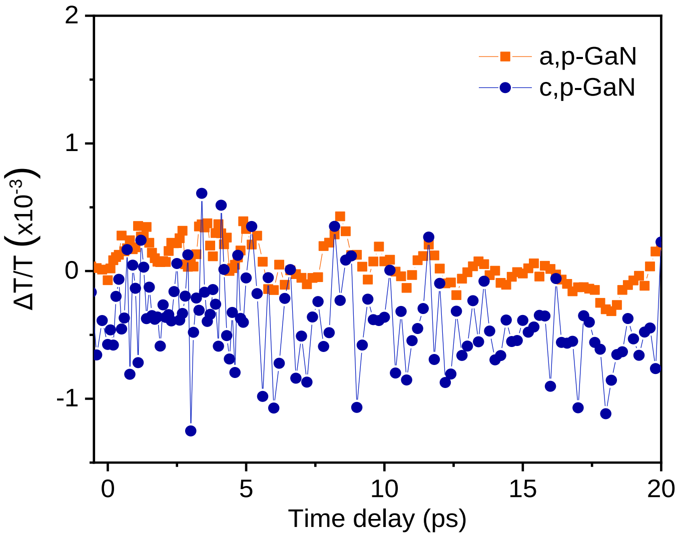

Fig. S6. Transient absorption spectra of the bare a,p-GaN and the bare c,p-GaN substrate probed at 650 nm. The samples were pumped at 620 nm with a power of 5 µW cm^{-2}.

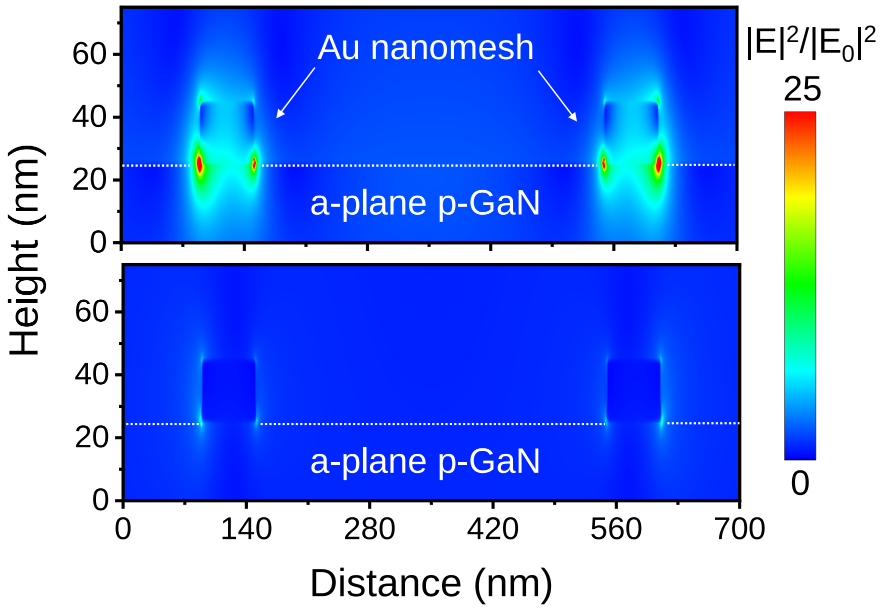

Fig. S7. FDTD simulated *E*-filed distribution on Au nanomesh/a,p-GaN structure at 660 nm (upper image) and 950 nm (lower image).

Supplementary Note 1. Calculation of external quantum efficiency (EQE) and internal quantum efficiency (IQE)

The EQE is defined by the following equation (*25*) :

$$EQE = \frac{number\ of\ \ collected\ electrons\ per\ second}{number\ of\ \ incident\ photons\ per\ second} = \frac{current \times 6.22 \times 10^{18}}{total\ energy / energy\ of\ \ one\ photon}$$

where we used a total energy of 1.97×10^{-11} J s^{-1} and single photon energies of 3.10×10^{-19} J. The IQE was calculated by normalizing the EQE with the absorption intensity (Fig. 1B). Since the measured photocurrent is primarily dominated by plasmonic hot-hole flux, the EQE represents the overall efficiency of converting incident photons into collected plasmonic hot holes within the Au/p-GaN Schottky architecture. In contrast, the IQE specifically evaluates the transport and collection efficiency of hot holes by isolating absorption efficiency effects.

Table S1. Summary of fit parameters acquired from the thermionic emission equation

	Au nanomesh /a,p-GaN	Au nanomesh /c,p-GaN	Units
Richardson constant (A*)	104	104	A/cm^2K
Temperature (T)	300	300	K
K_b	8.6×10^{-5}	8.6×10^{-5}	eV/K
KT/q	0.026	0.026	V
Area	7.96×10^{-5}	8.16×10^{-5}	cm^2
Ideality factor (η)	5.49	5.42	
Series resistance (R_S)	50	120	Mohm
Schottky barrier height (Φ_{SBH})	0.98	1.0	eV

Table S2. Elastic constants of the materials and calculated contact area.

Material	Poisson's ratio	Young's modulus (GPa)	Adhesion force (nN)	Contact area (nm^2)
PtIr (tip)	0.37	233	-	-
a,p-GaN	0.23	295	7.34	3.04
Net on Au/a,p-GaN	0.40	79	7.55	5.04
Edge on Au/a,p-GaN	0.40	79	9.06	5.69
c,p-GaN	0.23	295	11.2	4.03
Net on Au/c,p-GaN	0.40	79	5.52	4.08
Edge on Au/c,p-GaN	0.40	79	7.22	4.89
